# Ultrasonic Vibration Facilitates the Micro-Formability of a Zr-Based Metallic Glass

**DOI:** 10.3390/ma11122568

**Published:** 2018-12-17

**Authors:** Guangchao Han, Zhuo Peng, Linhong Xu, Ning Li

**Affiliations:** 1 School of Mechanical Engineering and Electronic Information, China University of Geosiences, Wuhan 430074, China; hgc009@cug.edu.cn (G.H.); 13237140699@163.com (Z.P.); xulinhong@cug.edu.cn (L.X.); 2 Shanxi Key Laboratory of Non-Traditional Machining, Xi’an Technological University, Xi’an 710032, China; 3 State Key Laboratory of Advanced Welding and Joining, Harbin Institute of Technology, Harbin 150001, China; 4 State Key Laboratory of Materials Processing and Die & Mold Technology, Huazhong University of Science and Technology, Wuhan 430074, China

**Keywords:** metallic glasses, thermoplastic microforming, ultrasonic vibration, formability

## Abstract

Thermoplastic microforming not only breaks through the bottleneck in the manufacture of metallic glasses, but also offers alluring prospects in microengineering applications. The microformability of metallic glasses decreases with a reduction in the mold size owing to the interfacial size effect, which seriously hinders their large-scale applications. Here, ultrasonic vibration was introduced as an effective method to improve the microformability of metallic glasses, owing to its capabilities of improving the material flow and reducing the interfacial friction. The results reveal that the microformability of supercooled Zr_35_Ti_30_Cu_8.25_Be_26.75_ metallic glasses is conspicuously enhanced by comparison with those under quasi-static loading. The more intriguing finding is that the microformability of the Zr-based metallic glasses can be further improved by tuning the amplitude of the ultrasonic vibration. The physical origin of the above scenario is understood, in depth, on the basis of ultrasonic vibration-assisted material flow, as demonstrated by the finite element method.

## 1. Introduction

Thermoplastic microforming (TPMF) has broken through the bottleneck in the manufacture of metallic glasses (MGs), providing an alternative way to fabricate MG parts/components with high precision and excellent mechanical properties, which offers alluring prospects of MGs in microengineering applications [1,2,3,4,5], while the microformability of MGs is seriously hindered by the interfacial effect and high viscosity in the supercooled liquid region (SCLR, a temperature window between glass transition temperature *T*_g_ and crystallization temperature *T_x_*). The low viscosity and the spatiotemporally homogeneous flow are also regarded as critical parameters that significantly affect the thermoplastic formability of MGs in the supercooled liquid state [6,7,8,9].

To improve the microformability of supercooled liquid MGs, processing parameters such as temperature and strain rate are usually considered as crucial factors, because these parameters determine the flow characteristics of the MGs. In our previous research [10], we revealed an inherent relationship between the thermoplastic formability and the flow characteristics, namely, Newtonian flow facilitates the forming capability, while thermoplastic forming in a non-Newtonian flow regime tends to be difficult. However, it is noted that the MGs would have a very short forming time window during the high-temperature forming process, which induces the risk of possible crystallization during plastic forming. To reduce the viscosity of MGs and avoid the possible crystallization, rapid temperature increases [11,12] and fast cooling [2] have been introduced to avoid the interaction with time–temperature–transformation (TTT) curve. On the other hand, in order to reduce the interfacial effect, the hot rolling method [13] and the thermoplastic blowing method [14] have been tried to reduce the physical contact area between MGs and the mold, while these methods are hard to be applied in the microforming of MGs. The method of adding lubricant has also been found to be very limited for reducing the interfacial friction. Therefore, a new method is urgent necessary to reduce the interfacial friction and lower the viscosity of MGs in the supercooled liquid region.

In recent years, vibration loading has been introduced to improve the microforming capacity of supercooled liquid MGs. It was found that low-frequency vibration (*f* ≤ 10 Hz) could effectively improve the plastic strain of MGs, owing to the vibration-assisted viscosity reduction and homogeneous flow [6]. Lateral extrusion experiments of Zr55 (Zr_55_Cu_30_Al_10_Ni_5_) MGs with low-frequency vibrations also revealed an enhanced microformability under vibration loading [15]. Ultrasonic vibration with high loading frequency of above 20 Hz has also been widely applied in metal microforming processes because of its superiority in enhancing formability via a reduction of the forming force and a decrease in friction at the interface [16,17,18], originating from the ultrasonic stress-softening effect, stress superposition effect, and periodic separation with ultrasonic vibration. Studies on ultrasonic vibration have been performed for shear formability of MGs [19]. This technique also exhibits potential applications in micro/nanoscale forming of MGs. By increasing loading frequency to about 20 KHz, Ma et al. [20,21] used high frequency ultrasonic beating method to fabricate micro- to macroscale structures, avoiding crystallization and oxidation of MGs, while the systemic investigation of ultrasonic vibration on microformability of metallic glasses is still lacking, and the underlying physical origin for ultrasonic-assisted thermoplastic forming of metallic glasses still remains unanswered.

In this work, ultrasonic vibrational loading was introduced as an innovative method to improve the thermoplastic microformability of Zr_35_Ti_30_Cu_8.25_Be_26.75_ MGs in a supercooled liquid state. It will be shown that the microformability of the supercooled liquid Zr-based MGs improves with increasing ultrasonic vibration amplitude, exhibiting an ultrasonic vibration-enhanced thermoplastic microformability. The physical mechanism of the phenomenon is rationalized in terms of the evolution of free volume concentration (*c*_f_) and flowing unit volume, which is further understood with assistance of finite-element-method (FEM) simulation. The present results provide an effective method to enhance the thermoplastic microformability of MGs.

## 2. Experimental Setup and Procedure

### 2.1. Materials

A Zr_35_Ti_30_Cu_8.25_Be_26.75_ (Zr35) MG system was selected for this research because of its excellent oxidation resistance, wide supercooled liquid region, and good glass-forming ability. Alloy cylinders with dimensions of ϕ 3 mm × 150 mm were fabricated by arc melting a mixture of pure Zr, Ti, Be, and Cu metals (purity > 99.5%) under a Ti-gettered argon atmosphere, followed by jet casting into a copper mold. The glassy structure of the as-cast alloy was verified by X-ray diffraction (XRD, Philips X’Pert Pro, Amsterdam, The Netherlands). The thermal response was determined by differential scanning calorimetry (DSC, TAQ2000, TA Instruments, New Castle, DE, USA) at a heating rate of 20 K·min^−1^, showing a glass transition temperature (*T*_g_) of 303.5 °C with a wide supercooled liquid region of 145 °C. The isothermal crystallization experiments revealed that the incubation time is more than 300 min at 370 °C [2,6,10,22]. The Zr35 samples with dimensions of ϕ 3 mm × 3 mm (strength of 1560 MPa) were sectioned from the bars, and the single factor experiments were completed for this research, such as ultrasonic power output, temperature, and forming velocity.

### 2.2. Ultrasonic Microextrusion System

**Figure 1:**
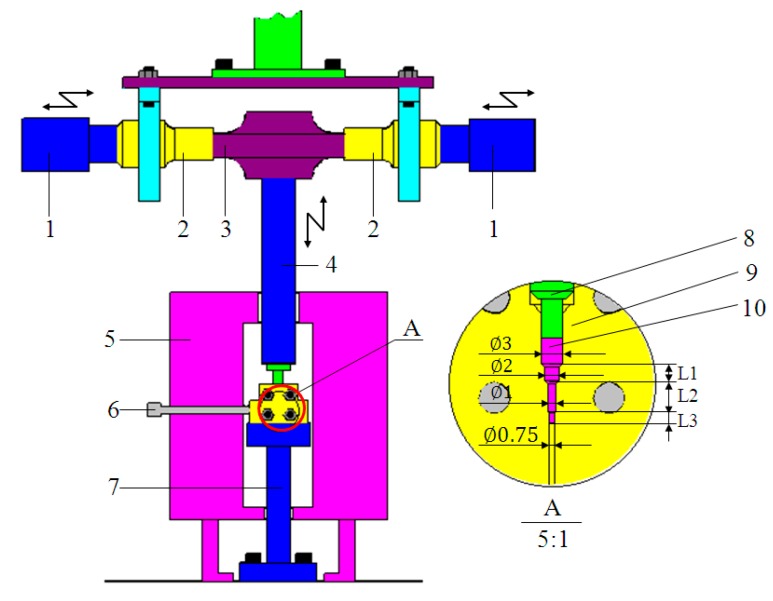
A schematic of the ultrasonic microextrusion system.

**Figure 2:**
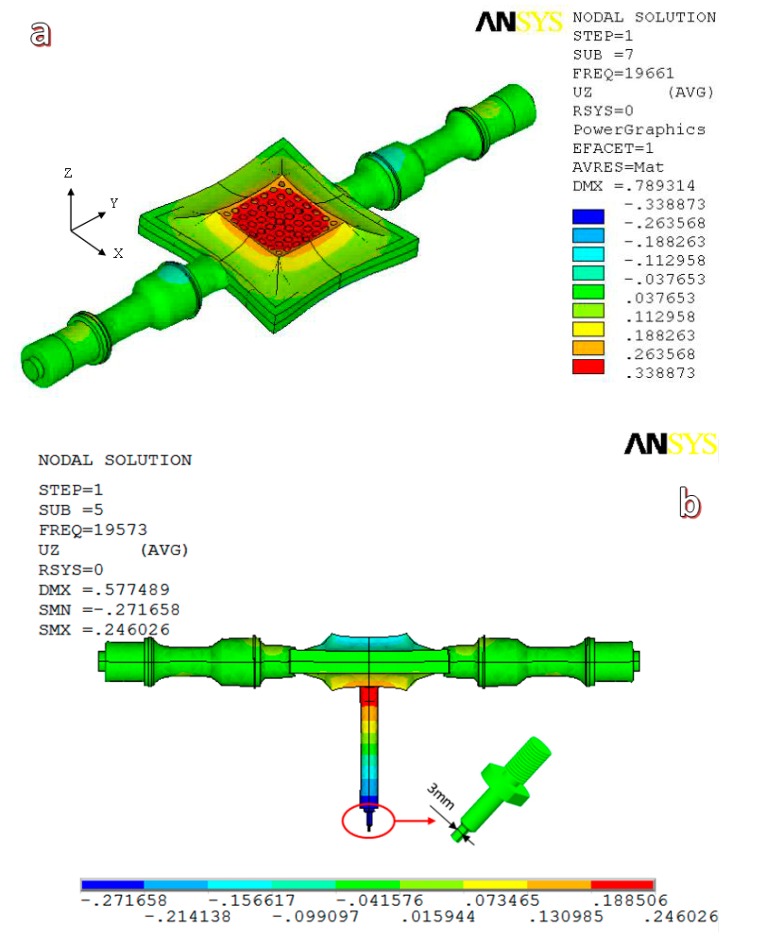
Models of the ultrasonic microextrusion systems in different situations: (**a**) model of the unloaded ultrasonic system; (**b**) model of the ultrasonic system loaded with a forming tool.

To meet the requirements of ultrasonic vibration and equipment installation, we designed and fabricated a 20 kHz ultrasonic vibration system by ourselves. The ultrasonic vibration system included a TJS-3000 ultrasonic generator, two YP5020-4D ultrasonic transducers, two ultrasonic step horns (which were all produced by Hangzhou Success Ultrasonic Equipment Co., Ltd., Hangzhou, China), and a special porous sonotrode. In contrast to standard ultrasonic devices with a vertical arrangement, we designed an ultrasonic vibration system with a horizontally symmetrical arrangement, wherein a cylindrical ultrasonic horn and a microforming indenter were connected in sequence to the surface center of the porous sonotrode, as shown in Figure 1. The porous sonotrode could convert the horizontal input vibrations of the ultrasonic transducers into vertical output vibrations [23], and the indenter could have vertical ultrasonic resonance vibrations with the sonotrode. Additionally, the ultrasonic vibration system could be easily mounted with a press machine through two flange support seats without the need for specially designed mounting structures. The ultrasonic vibration modes in the vertical direction of both the unloaded ultrasonic vibration system and the forming tool (cylindrical ultrasonic horn and indenter)-loaded system were simulated by ANSYS software (version 13.0), and the results are shown in Figure 2. The results show that the frequency of the ultrasonic system is reduced from 19,661 Hz (shown in Figure 2a) to 19,573 Hz (shown in Figure 2b) with the increase in loading (frequency tracking range of the TJS-3000 ultrasonic generator (Hangzhou Success Ultrasonic Equipment Co., Ltd., Hangzhou, China) is 20 ± 0.5 kHz), and the maximum ultrasonic amplitude can be obtained at the end of the indenter (shown in Figure 2b with blue color), which means that the ultrasonic system is insensitive to load fluctuations and is fit for vertical ultrasonic micro-extrusion processes.

In addition to the ultrasonic vibration system, the microextrusion device was composed of an M-4050 universal test machine produced by Shenzhen REGER (Shenzhen, China) and a KSY-6D-16 electric heating device produced by Wuhan Yahua Electric Furnace Co., Ltd. (Wuhan, China) (the temperature range was 50–1000 °C, and the temperature control precision was ±1 °C). The ultrasonic amplitude of the indenter was adjusted by the power output percentage of the ultrasonic generator, and the power output percentages in the experiments were set to 30%, 40%, 50%, and 60%. The corresponding ultrasonic amplitudes at the end of the indenter in the vertical direction were detected by a V100 laser vibration meter from RION Co., Ltd. (Tokyo, Japan) and were 12, 16, 20, and 24 μm, respectively. The mold and the indenter were made from H13 hot-work die steel (Guangzhou Hengwei Electromechanical Equipment Co., Ltd., Guangzhou, China). The two parts of the mold were clamped together by bolts, and the microextrusion mold cavity was composed of 3 circular subsection slots, *L*_1_, *L*_2_, and *L*_3_, whose diameters were 2, 1, and 0.75 mm, and lengths 2, 4, and 23.5 mm, respectively. The structure diagram of the mold and the indenter are also shown in Figure 1. The experimental temperature was set at 370, 380, and 390 °C in supercooled liquid region, while the microextrusion rate was set at 0.36 mm/min. The experimental microextrusion rate was set at 0.12, 0.24, 0.36 mm/min, while the temperature was set at 380 °C. During the ultrasonic microextrusion experiments, the mold was first heated to the setting temperature, and then the Zr35 sample was put into the mold, keeping the temperature for about 10 min, and the indenter contacted the sample with a pre-load of about 200 N. Then, the ultrasonic generator turned on, and the indenter started producing vertical ultrasonic vibration in addition to depressing the sample.

## 3. Results

### 3.1. Effect of Ultrasonic Power Output

**Figure 3:**
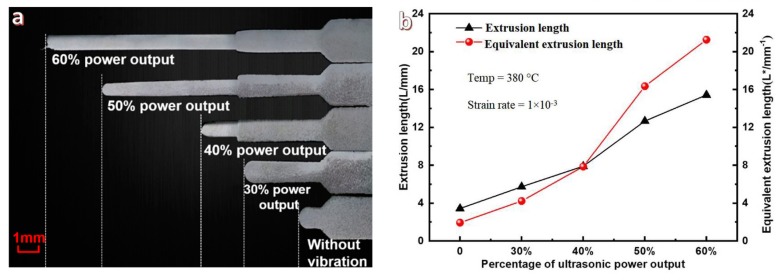
Microphotos of extruded Zr35 alloy (**a**) and recalculated microextrusion lengths of samples at various ultrasonic power outputs (**b**).

Figure 3 illustrates the microextrusion results under different ultrasonic power outputs (which corresponds to various ultrasonic vibration amplitudes for the indenter) and the filling lengths of the 3 different circular slots and the entire extrusion length are shown in Table 1. From Figure 3a, the extrusion length (*L*) increases with increasing ultrasonic power output, such as *L* is only 3.43 mm for the supercooled liquid MGs formed under static loading, while the value of *L* increases to 15.42 mm under ultrasonic loading with power output of 60%. The detailed data are summarized in Table 1, from which the Zr35 alloy can be pushed into the *L*_3_ circular slot (0.75 mm diameter) when the ultrasonic power output is greater than 30%, which indicates that a sufficiently large ultrasonic vibration amplitude is necessary for enhancing microfilling capacities of the Zr35 MG.

According to the previous research [24], the microforming ability of MGs on mold filling in the SCLR can be described as,

(1) $$P=32\eta \times v\frac{L}{d^{2}}$$

where *P* is the flow stress of the MGs, η is the apparent viscosity of a fluid, *v* is the fluid velocity, *d* is the diameter of the cylinder groove, and *L* is the fluid filling depth.

Accordingly, a similar approach is introduced to describe the microfilling capacity of MG at various sections with different sectional areas. $L_{i}^{*}$ is defined as the equivalent filling length of the different subsections, assuming that the whole equivalent microextrusion length is defined as L*, then,

(2) $$L^{*}=\overset{3}{\underset{i=1}{\sum }}L_{i}^{*}=\frac{L_{1}}{d_{1}^{2}}+\frac{L_{2}}{d_{2}^{2}}+\frac{L_{3}}{d_{3}^{2}}$$

where *L*_1_, *L*_2_, and *L*_3_ are the microfilling lengths of the different subsection slots shown in Figure 1, and *d*_1_, *d*_2_, and *d*_3_ are the corresponding diameters of these circular slots.

The extrusion lengths and the corresponding equivalent extrusion lengths are calculated, and summarized and described in Figure 3b. With increasing ultrasonic power output, it is clear that the equivalent extrusion lengths increase faster than the extrusion lengths. When the ultrasonic power output increased from 0% to 60%, the equivalent microextrusion lengths increased from 1.93 to 21.25 mm, which is 11 times longer than that of static loading, while the corresponding microextrusion length is just 4.5 times greater than that under static loading. Therefore, the equivalent extrusion length more objectively reflects the variation trends of the microfilling capacities of MGs at the micro- and nanoscales.

**Figure 4:**
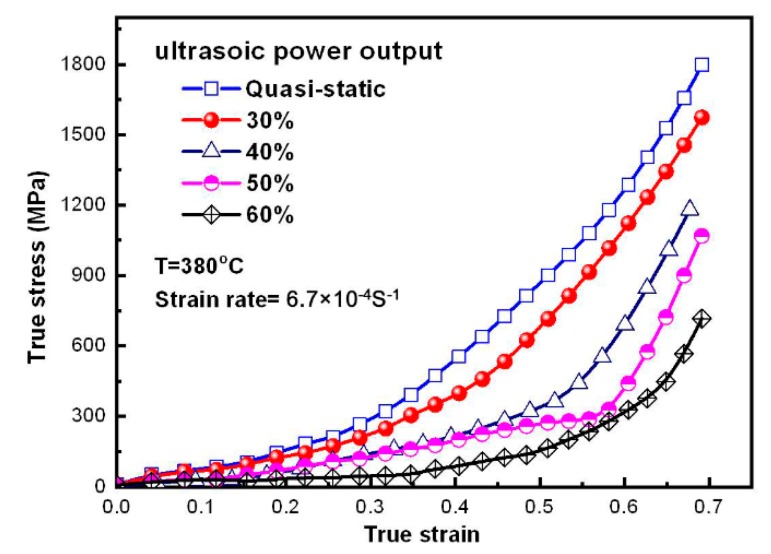
True stress–strain curves for different ultrasonic power outputs.

The true stress–strain curves of Zr35 MG extruded with different ultrasonic power outputs are depicted in Figure 4. The results show that the flow stress gradually reduces with increasing ultrasonic power output. Compared with the static loading process, the true stress is maximally decreased by 12.49%–60.17% when the ultrasonic power output increases from 30% to 60%.

### 3.2. Ultrasonic Loading under Various Temperatures

**Figure 5:**
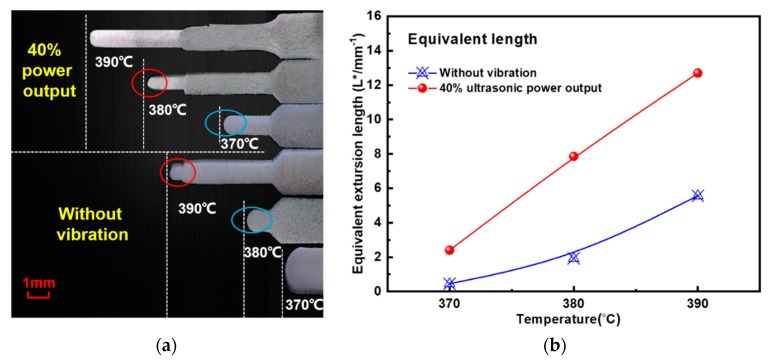
Ultrasonic microfilling length of Zr35 at different supercooled liquid temperatures: (**a**) microextrusion length (*L*); (**b**) equivalent extrusion length.

The ultrasonic microextrusion results with different supercooled liquid temperatures are shown in Figure 5, where the microextrusion rate is 0.36 mm/min and the ultrasonic power output is 40%. Figure 5a shows the microextrusion length (*L*) of Zr35 alloy at different supercooled liquid temperatures. Overall, the extrusion length increases with increasing supercooled liquid temperatures, wherein *L* with ultrasonic vibration at 370 or 380 °C is larger than that with static loading at 380 or 390 °C, which are marked with red and blue circles, respectively (the extrusion lengths for different temperatures are shown in Table 2). This phenomenon indicates that the ultrasonic vibration can improve the microfilling capacity of Zr35 MG similarly with temperature raising in the SCLR.

The equivalent extrusion lengths at different supercooled liquid temperatures are summarized in Figure 5b. The results reveal that the equivalent microextrusion lengths at 40% ultrasonic power output increase by 2, 5.83, and 7.13 respectively, compared with those of static loading under supercooled liquid temperatures of 370, 380, and 390 °C.

**Figure 6:**
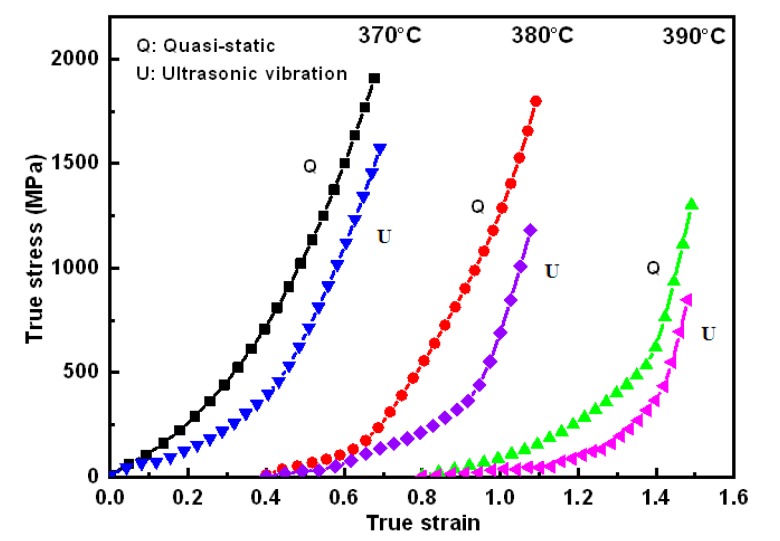
True stress–strain curves at different supercooled liquid temperatures.

The true stress–strain curves of Zr35 extruded at different supercooled liquid temperatures are shown in Figure 6. The results illustrate that the flow stress of Zr35 gradually reduces with increasing supercooled liquid temperatures. Compared with the static loading process, the true stress at 40% ultrasonic power output is maximally decreased by 23.67%, 34.36%, and 34.92%, respectively, when the supercooled liquid temperatures are 370, 380, and 390 °C.

### 3.3. Effect of Microextrusion Rate

**Figure 7:**
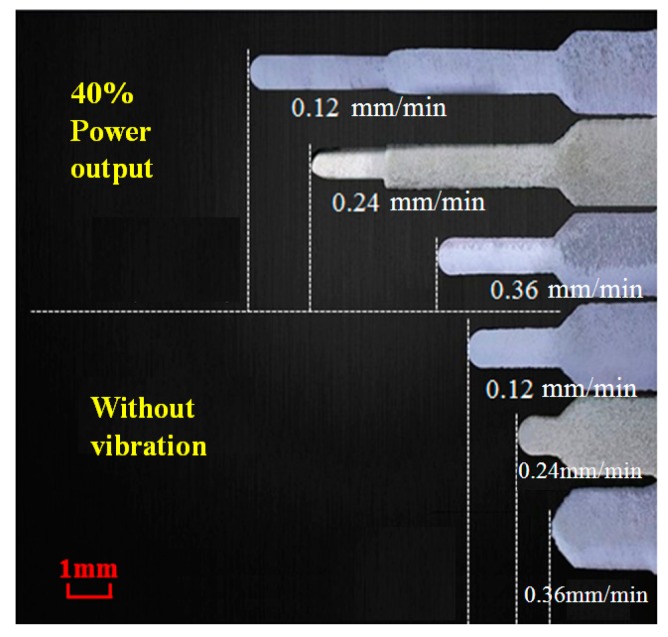
Ultrasonic extrusion length of Zr35 MG at various microextrusion rates.

Figure 7 illustrates the ultrasonic microextrusion lengths with various microextrusion rates, which is also summarized in Table 3, wherein the temperature is 380 °C and the ultrasonic power output is 40%. It is clear that the extrusion length reduces with the increasing of microextrusion speed. On the other hand, *L* is only 5.02 mm for the extrusion rate of 0.12 mm/min under static loading, while the value of *L* increases to 10.68 mm when the ultrasonic loading with power output of 40%, exhibiting an ultrasonic vibration dependence.

**Figure 8:**
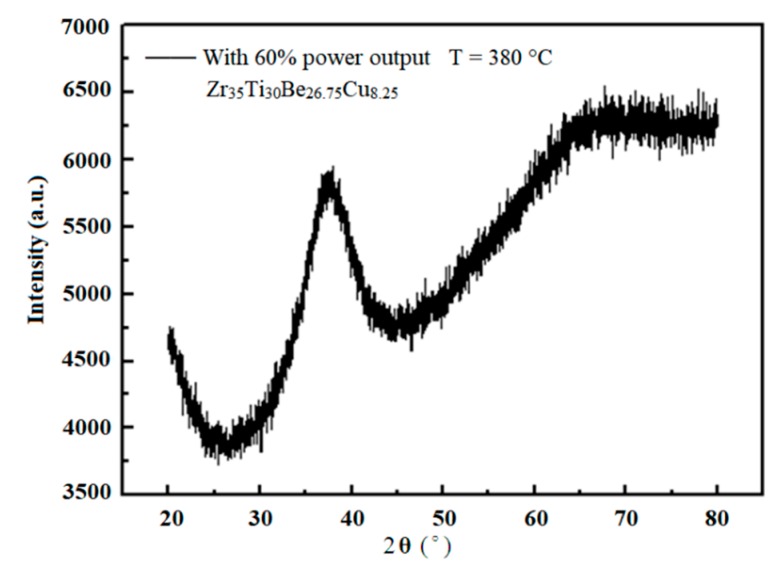
XRD results of ultrasonic uniaxial compressed Zr35 sample.

Finally, the glassy structure of the microformed Zr35 alloys were characterized with X-ray diffraction, and one of the representative results of 380 °C and 60% ultrasonic power output is illustrated in Figure 8, wherein only a wide dispersion peak appears, demonstrating that the Zr35 alloy remains as amorphous structure after ultrasonic uniaxial compression.

## 4. Discussion

The above phenomena can be analyzed in terms of ultrasonic energy transmission. Actually, it is very difficult to directly measure the ultrasonic vibration characteristics of MGs during the ultrasonic microextrusion process. Therefore, the ultrasonic vibration energy of the alloy obtained from the ultrasonic microextrusion process could be estimated according to the ultrasound energy density between the ultrasonic vibrating indenter and the Zr35 sample.

According to Yao [25], the coefficient of the sound power transmission from the H13 indenter to the Zr35 sample can be expressed as in Equation (3).

(3) $$\alpha_{t}=\frac{4\rho_{H13}c_{H13}\rho_{\mathrm{Zr}35}c_{\mathrm{Zr}35}}{(\rho_{H13}c_{H13}+\rho_{\mathrm{Zr}35}c_{\mathrm{Zr}35)}{}^{2}}$$

where *C*_H13_ and *C*_Zr35_ are the longitudinal vibration wave velocities of H13 die steel and MG samples, respectively. According to the material properties of H13 steel [26] and Zr35 [27], the value of *C*_Zr35_ is 2861.5 m/s and the value of *C*_H13_ is 6072 m/s. Therefore, the value of α_t_ is 0.739.

The sound energy density gained by the Zr35 sample can be calculated by Equation (4). The sound energy densities of the Zr35 samples at different ultrasonic power outputs are shown in Table 4.

(4) $$E_{n}=\frac{1}{2}\xi_{H13}^{2}\omega^{2}\rho_{H13}\alpha_{t}$$

*E*_n_ is the sound energy density of Zr35, ξ_H13_ is the vibration amplitude of the indenter, and ω is the excitation angular frequency (ω = 2π*f* = 125,600 rad/s).

Based on the ultrasonic softening and thermal activation theories [25], the reduction of the material flow stress caused by the ultrasonic vibrations can be attributed to the ultrasonic volume effect, which is comprised of the acoustic softening and stress superposition effects. The total flow stress reduction by the ultrasonic volume effect can be expressed as in Equation (5).

(5) $$\Delta \sigma_{s}=-K{\left(\alpha_{t}\xi \right)}^{n}-2\beta \hat{\tau }{\left(E_{n}/\hat{\tau }\right)}^{m}$$

The negative sign indicates stress reduction, β and *m* are experimental constants of the material (*m* > 0), $\hat{\tau }$ is the shear threshold of the material, which is equivalent to the shear strength of the material at absolute zero, α_t_ is the sound power transmission coefficient, *n* is the hardening index and the value is 1, and *K* is the material strength coefficient, which is the slope of the curve of stress reduction and ultrasonic amplitude caused by the stress superposition.

The parameters of β, *m*, $\hat{\tau }$, *K*, and *n* are intrinsic parameters of the material, so the shear stress drop, △σ_s_, is only related and proportional to the ultrasonic energy density, *E*_n_*,* and ultrasound amplitude, ξ. With an increase in the indenter’s ultrasonic vibration amplitude, the ultrasonic energy density, *E*_n_*,* absorbed by the Zr35 sample, should increase. Moreover, the value of the shear stress drop, △σ_s_*,* also increases, and the flow stress of Zr35 appears to decrease, which suggests that the ultrasonic volume effect on MGs is more obvious. Thus, the microforming capacity of Zr35 is greatly enhanced, and the equivalent whole extrusion length, L*, becomes longer.

The phenomenon of true stress–strain various with ultrasonic power output in Figure 4 can also be illustrated with the Equation (5). With increasing of power output, the ultrasonic vibration amplitude of the indenter is increased, while the stress superposition effect generated by the high frequency intermittent impact between the indenter and Zr35 sample is also strengthened, which possibly increases the decrease of the true stress of MGs. It is worth noting that previous research revealed that as for the metallic glasses, the vibration induced softening responsible for the reduction of stress [6]. On the other hand, part of the absorbed ultrasonic energy converted into heat [28], which can improve the material flow of Zr35 alloy in the SCLR. Furthermore, the ultrasonic vibration of the indenter can generate the ultrasonic softening effect in Zr35 [29], which will reduce the deformation resistance and improve the microfilling capacity of Zr35. Therefore, improvement in the microfilling capacity of MGs should be the combined effect of the ultrasonic softening effect and increasing temperature, and should be more obvious for MGs at lower temperatures. This is the reason why the ultrasonic microextrusion process can have a similar effect on improving the microforming capacity of MGs in the SCLR as increasing temperature. The increase in the microfilling capacity of Zr35 by ultrasonic vibration reaches a maximum value at 390 °C.

Furthermore, the phenomenon of true stress–strain with respect to supercooled liquid temperature in Figure 6 can be explained with free volume theory and viscosity variation of MGs. The higher temperature can lead to the increment of free volume concentration in the Zr35 and the decrease of viscosity, which facilitates the thermoplastic forming of Zr35 alloy. When the ultrasonic vibration is superimposed on the Zr35 samples, atomic diffusion increases and, therefore, there is a more conspicuous softening phenomenon.

## 5. FEM Analysis

In order to analyze the ultrasonic flow deformation mechanism of MGs in the SCLR, ABAQUS software was used to simulate the effects of the ultrasonic vibration amplitude on the flow deformation behavior of Zr35 during ultrasonic TPMF in the SCLR. A constitutive relation based on the free volume model was selected [30] and expressed as:

(6) $${\ln\tau }_{s}=\left(m+n\right)\left[\frac{W}{KT}+\left(\ln\gamma -{\ln\gamma }_{0}\right)\right]+\ln\left[s{\left(\frac{1}{4}\right)}^{m}\right]$$

where τ_s_ = 1/2σ is the shear stress, *m* = *n* = −1.31 × 10^−3^*T* + 1.00478 and s=21.6+866 {1+exp[(T−674)/13.5] are the temperature-dependent parameters, and $\gamma_{0}=5.07\;\times 10^{33}\;s^{-1}$ is the reference strain rate of Vitreloy-1 BMG (i.e., Zr_41.2_Ti_13.8_Cu_12.5_–Ni_10_Be_22.5_ BMG). The Zr-based MGs studied in the present work have a similar composition to the Vitreloy-1 BMG, and the above parameters can be used in the calculation.

To eliminate the influence of friction on the flow deformation behavior of the MGs, the uniaxial tensile process of the MGs was simulated to study the ultrasonic-assisted flow deformation mechanism of the MGs. The sample for the FEM simulations was set to be a cylindrical Zr35 sample with a diameter of 3 mm and a height of 10 mm. An axisymmetric two-dimensional simulation model was used for quasi-static analysis because of the symmetrical structural characteristics of the sample. The upper end of the sample was stretched, and the lower end was fixed. The tensile stroke was set at 4 mm.

In the ultrasonic uniaxial tensile simulation experiments of Zr35, the supercooled liquid temperature was set to 370 °C, the ultrasonic frequency was set to 20 kHz, and the ultrasonic amplitudes were set to 0, 12, and 24 μm, in turn. A 20 mm/s drawing speed was applied to the upper end of the sample, and a sinusoidal displacement was superimposed onto the lower end of the sample for simulating the ultrasonic vibration. When the vibration amplitude was 12 μm, the superimposed displacement could be calculated as follows:

(7) S=0.012sin125600t

**Figure 9:**
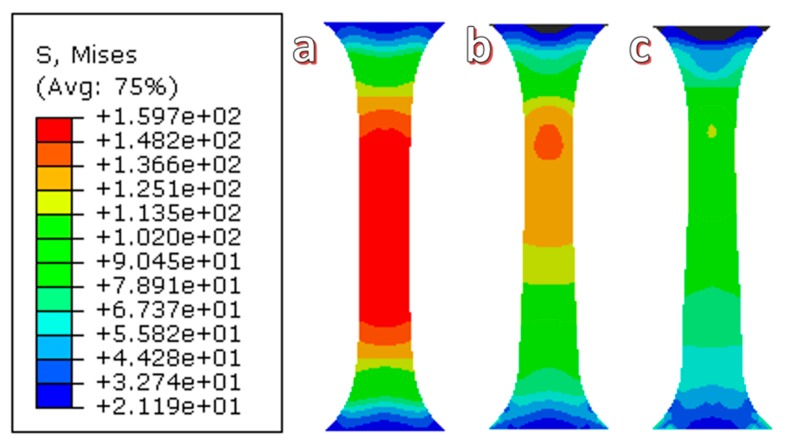
The distribution of the simulated equivalent stress the uniaxial tensile of Zr35 at different ultrasonic vibration amplitudes: (**a**) 0 μm; (**b**) 12 μm; and (**c**) 24 μm.

**Figure 10:**
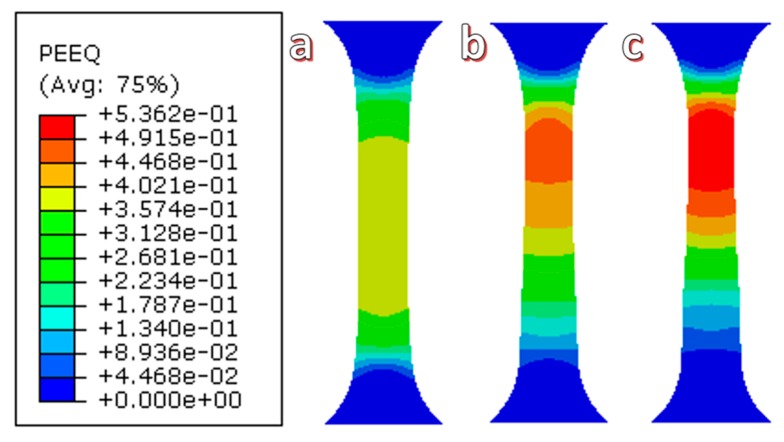
The distribution of the simulated equivalent strain of the uniaxial tensile of Zr35 at different ultrasonic vibration amplitudes: (**a**) 0 μm; (**b**) 12 μm; and (**c**) 24 μm.

The distribution of simulated equivalent stress of uniaxial tensile with ultrasonic vibration amplitudes of 0, 12, and 24 μm are shown in Figure 9a–c. The maximum equivalent stress at the different vibration amplitudes is 159.7, 138.2, and 114.0 MPa, respectively, which is a reduction of 13.5% and 28.6% with increasing ultrasonic amplitude. The distribution area of the maximum equivalent stress also decreased, which is shown in the red area of Figure 8. Additionally, the value of the equivalent strain is shown in Figure 10a–c, which was enhanced by the increasing ultrasonic vibration amplitude. The maximum equivalent strain increased by 16.74% and 33.38% when the ultrasonic vibration amplitude was increased to 12 and 24 μm, respectively. The maximum strain area exists in the upper and middle parts of the sample.

The reason for this phenomenon is mainly attributed to the ultrasonic softening effect of the material caused by the assisted ultrasonic vibration. In the process of ultrasonic plastic deformation, the stress field generated by the internal deformation of the material is superimposed over the periodic stress field of the ultrasonic vibration. With an increase in the ultrasonic amplitude, the effect of ultrasonic softening becomes more obvious, the plastic forming of the material becomes easier, and more free volume is generated in the plastic deformation process of the MGs. The higher free-volume concentration in the MGs means a lower viscosity for the MGs, together with smaller flow units in the sample under assisted ultrasonic vibration. The more homogeneous spatiotemporal distribution of flow units facilitates the thermoplastic microformability of the MGs, based on the above experimental results and theoretical analysis.

**Figure 11:**
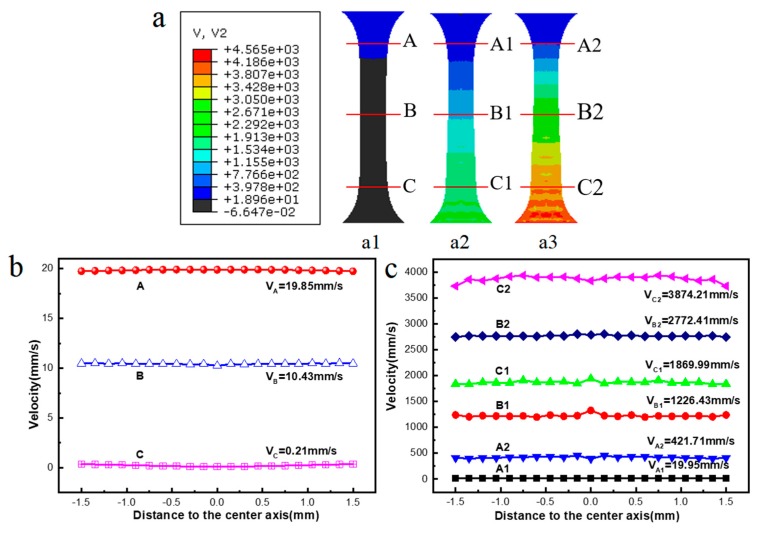
The simulated velocity distribution under three ultrasonic amplitudes by assuming a temperature of 370 °C: (**a1**) 0 μm; (**a2**) 15 μm; and (**a3**) 30 μm. (**b**,**c**) present the corresponding speed distribution at various positions.

The profiles of the corresponding velocity distributions obtained from the above FEM simulation results are shown in Figure 11. The results show that the velocity is distributed heterogeneously along the uniaxial tensile direction due to the unidirectional stress. To further distinguish differences in the material flow during the ultrasonic uniaxial tensile process at different vibration amplitudes (i.e., 0, 15, and 30 μm), the velocities (*V*) at positions A–C (with an amplitude of 0 μm), A1–C1 (with an amplitude of 15 μm) and A2–C2 (with an amplitude of 30 μm) are calculated and compared, as shown in Figure 11a. As delineated in Figure 11b,c, the flowing velocity has a value ranging from 19.85 to 1869.99 mm/s at positions A1–C1, and a value ranging from 421.71 to 3874.21 mm/s at positions A2–C2, which are two orders of magnitude greater than the velocities with vibration in the A–C areas (0.21–19.85 mm/s). This indicates that a larger ultrasonic vibration amplitude corresponds to a greater change in the flow rate of the amorphous alloy.

Kim proposed a free-volume constitutive model to characterize the internal atom changes in amorphous alloys by studying the deformation behavior of amorphous alloys [30]. The relationship between free volume and strain rate is expressed in Equation (8).

(8) $$v_{f}^{*}={\left[\frac{1}{v_{fe}}-\left(c\ln\gamma +l\right)\right]}^{-1}$$

Here, $v_{f}^{*}$ is the steady state free volume, *v*_fe_ = (*T* − *T*_0_)/*DT*_0_ is the balanced free volume, and *c* and *l* are temperature-related parameters.

From the above Equation (8), it can be concluded that an increase in the strain rate will lead to an increase in the steady-state free volume and a decrease in the viscosity of the amorphous alloy, and the occurrence of plastic deformation will also be easier.

In general, higher ultrasonic vibration amplitudes can achieve higher flow velocities and easier material flow of Zr35 MGs in the SCLR. Thus, the microplastic forming capacity of Zr35 can be gradually improved by increasing the vibration amplitude, which is in agreement with the analysis of the equivalent stress and strain above.

## 6. Conclusions

In this work, the ultrasonic vibration was introduced to the microextrusion processing of MGs. The ultrasonic microformability of Zr35 in the SCLR was studied with experiments and FEM. The conclusions are as follows.

- Ultrasonic vibration is an effective method to improve the thermoplastic microformability of MGs by superimposing the assisted ultrasonic vibration onto the microextrusion tool.
- With increasing ultrasonic power output, the equivalent microfilling length of Zr35 can be increased by 11-fold, and the true stress can be decreased by 60.17%.
- Sufficient ultrasonic power output (>30%), which means a sufficiently large ultrasonic vibration amplitude of the microextrusion tool, is the essential condition to obtain better microformability of Zr35, which can be successfully packed into a circular slot with a 0.75 mm diameter.
- Ultrasonic vibration of the microextrusion tool can produce a similar effect as temperature increase on improving the microformability of Zr35 in the SCLR.
- Larger ultrasonic vibration amplitudes of the tool can generate more free volume in the plastic microforming process of Zr35 MGs, which can then obtain a higher flow velocity and easier material flow.

## Figures and Tables

**Table 1:** Microextrusion length of Zr35 alloy at various ultrasonic output powers.

Ultrasonic Output Power	First Section Slot Length (*L*_1_) (mm)	Second Section Slot Length (*L*_2_) (mm)	Third Section Slot Length (*L*_3_) (mm)	Whole Extrusion Length (*L* = *L*_1_ + *L*_2_ + *L*_3_) (mm)
0	2	1.43	0	3.43
30%	2	3.72	0	5.72
40%	2	4	1.89	7.89
50%	2	4	6.66	12.66
60%	2	4	9.42	15.42

**Table 2:** Extrusion length of Zr35 at different supercooled liquid temperatures.

Extrusion Length and Temperature	Without Ultrasonic Vibration			With 40% Ultrasonic Output Power		
Supercooled liquid temperature (°C)	370	380	390	370	380	390
First section length (*L*_1_/mm)	1.81	2.00	2.00	2.00	2.00	2.00
Second section length (*L*_2_/mm)	0	1.43	4.00	1.90	4.00	4.00
Third section length (*L*_3_/mm)	0	0	0.60	0	1.89	4.61
Whole extrusion length (*L* = *L*_1_ + *L*_2_ + *L*_3_/mm)	1.81	3.43	6.60	3.90	7.89	10.61

**Table 3:** Extrusion length of Zr35 at different microextrusion rates.

Extrusion Rate and Length	Without Ultrasonic Vibration			With 40% Ultrasonic Output Power		
Microextrusion rate (mm/min)	0.12	0.24	0.36	0.12	0.24	0.36
First section length (*L*_1_/mm)	2.00	2.00	2.00	2.00	2.00	2.00
Second section length (*L*_2_/mm)	3.02	1.93	0.78	4.00	4.00	3.93
Third section length (*L*_3_/mm)	0	0	0	4.68	2.39	0
Whole extrusion length (*L* = *L*_1_ + *L*_2_ + *L*_3_/mm)	5.02	3.93	2.78	10.68	8.39	5.93

**Table 4:** The ultrasonic energy densities and amplitudes at different ultrasonic power outputs.

Ultrasonic Power Output	Ultrasonic Amplitudes ξ_H13_ (μm)	Energy Density *E*_n_ (kJ/m^3^)
0	0	0
30%	12	7.41
40%	16	13.173
50%	20	20.583
60%	24	29.64
